# Supplementary material for: Mosquito-Borne Diseases and Their Control Strategies: An Overview Focused on Green Synthesized Plant-Based Metallic Nanoparticles
Source: Insects. 2023 Feb 23;14(3):221. doi: 10.3390/insects14030221 (PMC10059804; doi:10.3390/insects14030221)
Supplement: Supplementary file 1 [file insects-14-00221-s001.zip › insects-2095625-supplementary.pdf]

Table S1. Green metallic nanoparticles with mosquitocidal properties.

	Materials		Operative conditions for synthesis	Shape and size	Activity and species	Origin of mosquitoes	LC_{50}[a]	Ref
	Plants	Metal precursors						
1.	*Sargassum muticum* leaves (aqueous extract 5%)	$AgNO_3$ 1mM	Ratio NM[b] Incubation time NM Temperature: 95 °C	Spherical, 43–79nm (SEM, EDX)[c,d]	Larvicidal and pupicidal (*Ae. aegypti, An. stephensi* and *Cx. quinquefasciatus*)	Laboratory strains	28.10 26.09 30.16	[203]
2.	*Plumbago auriculata* Lam leaves (aqueous extract 10%)	$AgNO_3$ 1mM	Ratio 1:10 Incubation time: 24h Temperature: 25°±2°C	Spherical and hexagonal, < 50 nm (TEM, FE-SEM, EDX)[e,f]	Larvicidal (*Ae. aegypti* and *Cx. quinquefasciatus*)	Wild population	45.1 41.1	[233]
3.	*Blumea mollis*	$AgNO_3$ NM	Ratio NM Incubation time NM Temperature NM	23–31 nm (SEM)	Larvicidal (*An. subpictus, Cx. vishnui* and *Ae. vittatus*)	Laboratory strains	18.17 23.45 21.82	[234]
4.	*Phyllanthus niruri* leaves (aqueous extract 10%)	$AgNO_3$ 1mM	Ratio NM Incubation Time NM Temperature NM	Spherical, 20– 60 nm (SEM)	Larvicidal and adulticidal (*Ae. aegypti*)	Laboratory strains	210.53 3.9-13.04	[235]
5.	*Morinda tinctoria* leaves (acetone extract 10%)	AgNO3 1mM	Ratio 1:9 Incubation: 1h Temperature: room	Spherical, 60– 95 nm (TEM)	Larvicidal (*Cx. quinquefasciatus*)	Laboratory strains	1.44	[238]
6.	*Ficus religiosa,* leaves (aqueous extract 20%)	$TiO(OH)_2$ 5 mM	Ratio 2:8 Incubation time: 4h Temperature: 50°C	Spherical, 70.29–84.93 nm (TEM)	Larvicidal (*An. stephensi*)	Laboratory strains	50	[239]
7.	*Ricinus communis* leaves (acetone extract 20%)	$AgNO_3$ 1 mM	Ratio 9:1 Incubation time: 1h Temperature 40°C pH 8 (NaOH 0,1%)	NM	Larvicidal (*Ae. albopictus*)	Laboratory strains	49.43–149.58	[243]
8.	*Cocoa pod husk* fruits (aqueous extract 10%)	$AgNO_3$ 1 mM	Ratio 40:1 Incubation time: NM Temperature: 30±2°C	Spherical, 4-32 nm (TEM)	Larvicidal (*An. spp.*)	Wild populations	43.52	[244]
9.	*Nerium oleander* leaves (aqueous extract 10%)	$AgNO_3$ 1mM	Ratio NM Incubation Time NM Temperature: room	Spherical, 20– 35 nm (SEM)	Larvicidal (*An. stephensi*)	Wild populations	33.99	[245]
10.	*Leucas aspera* leaves (methanol extract 33%)	$AgNO_3$ 1mM	Ratio NM Incubation time NM Temperature: room	Spherical and cubical, 30– 37 nm (EDS, SEM)[g]	Larvicidal and pupicidal (*Ae. aegypti* and *Ae. stephensi*)	NM	NM	[246]
11.	*Ambrosia arborescens* leaves (aqueous extract 5%	$AgNO_3$ 1mM	Ratio 1:10 Incubation time: 50 min Temperature: 50° C pH: 8	Spherical, 14±6 nm (TEM)	Larvicidal *(Ae. aegypti)*	Laboratory strains	0.28	[247]
12.	*Solanaum lycopersicum* leaf (aqueous extract 10%)	$Zn(NO_3)_2$ NM	Ratio NM Incubation time: 2 h Temperature: 60°C	Rod-like, pellet-like shape < 100 nm (SEM)	Larvicidal *(Ae. aegypti)*	NM	9.31	[250]
13.	*Eucalyptis grandis* bark (aqueous extract 4%)	$AgNO_3$ 1mM	Ratio NM Incubation Time NM Temperature: room	NM	Larvicidal (*Ae. aegypti* and *Cx. quinquefasciatus*)	Laboratory strains	42 35	[252]
14.	*Ulva lactuca* leaves (ethanol extract	$AgNO_3$ 1mM	Ratio NM	Crystalline and cubical, 20–	Larvicidal and pupicidal	Wild	5.26	[253]

	33%)		Incubation Time NM<br>Temperature NM	35 nm (SEM)	(*An. stephensi*)	populations		
15.	*Hugonia mystax*<br>(aqueous extract 10%)	$AgNO_3$ 1mM	Ratio 3:22<br>Incubation time: 10 min<br>Temperature: room	Spherical, 40– 90 nm (TEM, EDX, SEM)	Larvicidal<br>(*An. stephensi, Ae. aegypti* and *Cx. quinquefasciatus*)	Laboratory strains	162.66<br>181.75<br>199.47	[254]
16.	*Hypnea musciformis*<br>Seaweed (aqueous extract 10%)	$AgNO_3$ 1mM	NM	Cubic, 40–65 nm (SEM)	Larvicidal and pupicidal<br>*(Ae.aegypti)*	Wild populations	18.14–38.23<br>24.5–38.23	[259]
17.	*Pteridium aquilinum* leaves<br>(aqueous extract 10%)	$AgNO_3$ 1mM	Ratio 3:1<br>Incubation time NM<br>Temperature: Room temperature	Spherical, 35– 65 nm (SEM)	Larvicidal and pupicidal<br>(*An. stephensi*)	Wild populations	18.45	[260]
18.	*Dicranopteris linearis*<br>Leaves (aqueous extract 10%)	$AgNO_3$ 1mM	NM	Spherical, 40– 60 m (SEM)	Larvicidal and pupicidal<br>*(Ae. aegypti)*	Laboratory strains	18.91 (larva)<br>29.33 (pupa)	[261]
19.	*Rubus ellipticus*<br>Leaves (aqueous extract 50%)	$AgNO_3$ 1mM	Ratio 12/88<br>Incubation time 10 min<br>Temperature NM	Spherical, 30 nm (TEM)	Larvicidal, ovicidal and adulticidal<br>*(An. stephensi, Ae. aegypti* and *Cx. quinquefasciatus)*	NM	12.50<br>13.83<br>15.09	[262]
20.	*Gracilaria firma*<br>Seaweed (aqueous extract 10%)	$AgNO_3$ 1mM	NM	Spherical, 12–200 nm (TEM)	Larvicidal and pupicidal<br>*(Ae. aegypti)*	Laboratory strains	25.89–351.41	[268]
21.	*Pergularia daemia*<br>Latex	$AgNO_3$ 1mM	Ratio 1:100<br>Incubation time: 10 min<br>Temperature NM	Spherical, 44– 245 nm (TEM)	Larvicidal<br>*(Ae. aegypti* and *An. stephensi)*	Wild populations	4.39–6.18<br>4.41–6.47	[275]
22.	*Plumeria rubra*<br>Latex	$AgNO_3$ 1mM	Ratio 1:100<br>Incubation time: 10 min<br>Temperature NM	Spherical, 32– 220 mn (TEM)	Larvicidal<br>*(Ae. aegypti* and *An. stephensi)*	Wild populations	0.31	[278]
23.	*Drypetes roxburghii,* fruits (aqueous extract 5%)	$AgNO_3$ 25 mM	Ratio 20:25<br>Incubation time: 8h<br>Temperature 50°C	Spherical or polyhedral, 10–35 nm (HR-TEM)[h]	Larvicidal<br>(*An. stephensi* and *Cx. quinquefasciatus*)	Wild populations	0.80–1.14<br>0.92–1.40	[281]
24.	*Vinca rosea*<br>Leaves (aqueous extract 10%)	$AgNO_3$ 1mM	Ratio 88:12<br>Incubation time: NM<br>Temperature NM	Spherical, 25– 47 nm (TEM)	Larvicidal<br>*(An. stephensi* and *Cx. quinquefasciatus)*	Wild populations	12.47–16.84<br>43.80	[282]
25.	*Melia azedarach*<br>leaves (ethyl acetate, hexane, chloroform and methanol extracts)	$AgNO_3$ 0.5 mM	Ratio 13:87	Spherical, 3– 31 nm (HR-TEM)	Larvicidal<br>(*Ae. aegypti* and *Cx. quinquefasciatus*)		12.37–23.21	[283]
26.	*Tinospora cordifolia*<br>Leaves (aqueous extract 5%)	$AgNO_3$ 1mM	Ratio 1:9<br>Incubation time: 10 min<br>Temperature NM	55–80 nm (SEM, TEM)	larvicidal<br>*(An. subpictus* and *Cx. quinquefasciatus)*	Wild populations	12.46<br>6.43<br>6.96	[287]
27.	*Solanum mammosum* L., fruits (aqueous extract 5%)	$AgNO_3$ 1mM	Ratio NM<br>Incubation time: 30 min<br>Temperature NM[b]<br>pH: 9 (NaOH 0,1 M)	Cubic, 10– 14 nm (TEM)	Larvicidal<br>(*Ae. aegypti L.*)	Wild populations	0.06 ± 1.02	[291]
28.	*Murraya koenigii,* leaves (methanolic extract 33,3%)	$AgNO_3$ 1mM	Ratio NM<br>Incubation Time NM<br>Temperature NM	Spherical and cubic, 20– 35 nm<br>(TEM)	Larvicidal<br>(*An. stephensi* and *Ae. aegypti*)	Wild populations	279.33–700.16<br>314.29–774.01	[292]
29.	*Tinospora cordifolia*<br>leaves (aqueous extract 5%)	$AgNO_3$ 1 mM	Ratio 9:1<br>Incubation time: 10 min	Spherical, 55–80 nm (SEM)	Larvicidal<br>(*An. subpictus* and *Cx.*	Wild populations	6.43<br>6.96	[293]

			Temperature NM		*quinquefasciatus*)			
30.	*Aquilaria sinensis,* essential oil	$AgNO_3$ 2mM	Ratio NM<br>Incubation time NM<br>Temperature NM<br>pH 7 (NaOH 0,1 M)	Spherical, 15– 55 nm (SEM)	Larvicidal and pupicidal<br>(*Ae. albopictus*)	Laboratory strains	44.23–166	[294]
31.	*Pogostemon cablin,* essential oil			Spherical, 16– 87 nm (SEM)			32.49–90.05	
32.	*Avicennia marina*<br>leaves (aqueous extract 10%)	$AgNO_3$ NM	Ratio 9:1<br>Incubation time: 10 min<br>Temperature NM	Spherical, 7,67– 8,34 nm (SEM)	Larvicidal<br>(*Ae. aegypti* and *An. stephensi*)	Laboratory strains	4.37<br>7.41	[295]
33.	*Azadirachta indica* leaves (aqueous extract 20%)	$AgNO_3$ 1 mM	Ratio NM<br>Temperature 70°C<br>Incubation time: 10 min	Spherical, 12– 24 nm (SEM)	Larvicidal<br>(*Ae. aegypti*)	Wild populations	1.25	[296]
34.	*Citrullus colocynthis* fruits (aqueous extract 25%)	$AgNO_3$ 5 mM	Ratio 20:1, 20:2 and 20:4<br>Temperature: 95°C<br>Incubation time: 20 min	Spherical, 20–36 nm (SEM)	Larvicidal<br>(*Ae. aegypti*)	Wild populations	0.3	[296]
35.	*Cyprus rotundas* roots (aqueous extract 2%)	$AgNO_3$ 10 mM	Ratio 2:1<br>Temperature: 50°C<br>Incubation time: 2h	Spherical, 5–20 nm (HR-TEM)	Larvicidal<br>(*Ae. albopictus, Ae. aegypti, Cx. quinquefasciatus* and *An. Stephensi*)	Laboratory strains	0.001–0.02	[297]
36.	*Annona glabra*<br>leaves (aqueous extract 20%)	$AgNO_3$ 1 mM	Ratio 10:1 and 10:2<br>Incubation time: 3h<br>Temperature NM	Spherical, 10– 100 nm and 100–1000 nm (SEM)	Larvicidal<br>(*Ae. aegypti* and *Ae. albopictus*)	NM	2.43–5.9<br>2.51–3.02	[298]
37.	*Cleistanthus collinus*<br>leaves (aqueous extract 25%)	$AgNO_3$ 1 mM	Ratio 5:9<br>Incubation Time NM<br>Temperature NM	30–50 nm (SEM)	Larvicidal<br>(*Ae. aegypti*)	NM	NM	[299]
38.	*Achyranthes aspera*<br>stem (aqueous extract 10%)	$AgNO_3$ 3 mM	Ratio NM<br>Temperature 85-95°C<br>Incubation time 30 min	Spherical, 25 nm (SEM)<br>Spherical, 30 nm (TEM)	Larvicidal<br>(*Ae. aegypti*)	NM	4.28–26.69	[300]
39.	*Pimenta dioica* leaves (aqueous extract 5%)	$AgNO_3$ 1 mM	Ratio 1:9<br>Incubation time: 20 min<br>Temperature: 60°C	spherical and triangular, 25–60 nm (SEM)<br>20–40 nm (TEM)	Larvicidal<br>(*An. stephensi, Cx. quinquefasciatus* and *Ae. aegypti*)	NM	3.27<br>5.37<br>2.61	[301]
40.	*Leucas aspera*<br>leaves (aqueous extract 10%)	$AgNO_3$ 1 mM	Ratio 2:98<br>Incubation time: 1h<br>Temperature 28°C	Spherical, 22–43 nm (FE-SEM)	Larvicidal<br>(*Ae. aegypti, An. stephensi,* and *Cx. quinquefasciatus*)	Wild populations	4.02<br>4.69<br>5.06	[302]
41.	*Hyptis suaveolens*<br>leaves (aqueous extract 10%)	$AgNO_3$ 1 mM		Spherical, 19–35 nm (FE-SEM)			4.63<br>4.04<br>3.52	
42.	*Carica papaya*<br>Latex 5% (chloroform, methanol, and water)	$AgNO_3$ NM	Ratio NM<br>Incubation Time NM<br>Temperature NM	Spherical, 12 ± 6 nm (TEM)	*Larvicidal*<br>(*Ae. aegypti* and *Cx. quinquefasciatus*)	Laboratory strains	1.58–2.47	[303]
43.	*Excoecaria agallocha* leaves (aqueous extract 5%)	$AgNO_3$ 1mM	Ratio 1:19<br>Incubation time NM<br>Temperature NM	Spherical, 18–50 nm (HR-TEM)	Lavicidal<br>(*Ae. aegypti*)	Wild populations	4.65	[304]
44.	*Malus domestica*<br>fruits (aqueous extract 50%)	$AgNO_3$ 0,1M	Ratio 1:1<br>Incubation time NM<br>Temperature NM	Spherical,50–120 nm (FE-SEM)	Larvicidal<br>(*Ae. aegypti*)	NM	15.76	[305]

45.	*Pedilanthus tithymaloides* leaves (aqueous extract 10%)	$AgNO_3$ 1mM	Ratio 1:9<br>Incubation time NM<br>Temperature: room	Spherical 15– 30 nm (TEM)	Larvicidal and pupicidal (*Ae. aegypti*)	Laboratory strains	0.086 %	[306]
46.	*Delphinium denudatum* Root (aqueous extract 10%)	$AgNO_3$ 1mM	Ratio 1:20<br>Incubation time: 2 h<br>Temperature NM	Spherical, 85 nm (FE-SEM)	Larvicidal *(Ae. aegypti)*	Laboratory strains	96	[307]
47.	*Menyanthes trifoliata* leaves (aqueous extract 1%)	$AgNO_3$ 1mM	Ratio 1:9<br>Incubation: 24h<br>Temperature: room<br>pH acid	Spherical, 40– 60 nm (SEM)	Larvicidal and pupicidal (*Ae. aegypti*)	Wild populations	NM	[308]
48.	*Acalypha fruticosa,* leaves (aqueous extract 10%)	$AgNO_3$ 1mM<br>$HAuCl_4$ 1mM	Ratio NM<br>Incubation time: 72 h<br>Temperature NM	Spherical, 100 nm (AgNPs)<br>Spherical, 90 nm (AuNPs)	Larvicidal (*Cx. pipiens*)	Wild populations	52.86	[309]
49.	*Suaeda maritime* Leaves (aqueous extract 10%)	$AgNO_3$ 1mM	NM	Spherical, 20– 60 nm (SEM)	Larvicidal pupicidal and ovicidal *(Ae. aegypti)*	Laboratory strains	8.67–17.98<br>20.94–46.90	[310]
50.	*Carissa carandas* Leaves (aqueous extract 10%)	$AgNO_3$ 1mM	Ratio 88:12<br>Incubation time: 10 min<br>Temperature NM	Spherical, 1.6–7.4 nm (AFM)[i]	Ovicidal and larvicidal *(An. stephensi, Ae. aegypti* and *Cx. quinquefasciatus)*	Laboratory strains	14.33<br>15.69<br>16.95	[311]
51.	*Aganosma cymosa* Leaves (aqueous extract 10%)	$AgNO_3$ 1mM	Ratio 88:12<br>Incubation time: 10 min<br>Temperature NM	Spherical, 1– 16.5 nm (AFM)	Ovicidal and larvicidal *(An. stephensi, Ae. aegypti* and *Cx. quinquefasciatus)*	Laboratory strains	12.45<br>13.58<br>14.79	[312]
52.	*Bougainvillea glabra* Leaves (aqueous extract 10%)	$AgNO_3$ 1mM	NM	Spherical, 20 nm (TEM)	Larvicidal, pupicidal, ovicidal and adulticidal *(Cx. tritaeniorhynchus)*	Wild populations	7.77-19.44	[313]
53.	*Ventilago maderaspatana* Leaves (aqueous extract 10%)	$AgNO_3$ 1mM	Ratio 12:88<br>Incubation time: 10 min<br>Temperature NM	Spherical, 4.6–5 nm (AFM, SEM)	Larvicidal, ovicidal and adulticidal *(An. stephensi, Ae. aegypti*, and *Cx. quinquefasciatus)*	Laboratory strains	41.19<br>44.85<br>48.94	[314]
54.	*Naregamia alata* Leaves (aqueous extract 10%)	$AgNO_3$ 1mM	Ratio 12:88<br>Incubation time: 10 min<br>Temperature NM	Spherical, 5– 350nm (AFM, SEM, TEM)	Larvicidal, ovicidal and adulticidal *(An. stephensi, Ae. aegypti*, and *Cx. quinquefasciatus)*	Laboratory strains	31.60<br>34.31<br>37.52	[315]
55.	*Juniperus procera* Leaves (methanol extract 20%)	$AgNO_3$ 1mM	Ratio 1:1<br>Incubation time: 10 min<br>Temperature: room	Spherical, 230 nm (SEM)	Larvicidal *(Ae. aegypti)*	Laboratory strains	30.49	[316]
56.	*Feronia elephantum* Leaves (aqueous extract 10%)	$AgNO_3$ 1mM	Ratio 12:88<br>Incubation time 10 min<br>Temperature NM	Triangular, pentagonal and hexagonal, 20– 60 nm (SEM)	Larvicidal *(Cx. quinquefasciatus, An. stephensi,* and *Ae. aegypti)*	Laboratory strains	14.19<br>11.56<br>13.13	[317]
57.	*Feronia elephantum* Leaves (aqueous extract 10%)	$AgNO_3$ 1mM	Ratio 12:88<br>Incubation time: 10 min<br>Temperature NM	Spherical and triangle, 18–45 nm (TEM)	Adulticidal *(An. stephensi, Ae. aegypti, and C. quinquefasciatus)*	Laboratory strains	18.04<br>20.40<br>21.80	[318]

58.	*Heliotropium indicum* Leaves (aqueous extract 10%)	$AgNO_3$ 1mM	Ratio 12:88 Incubation time: 10 min Temperature NM	Spherical, triangle and decahedral, 18–45 nm (TEM)	*Larvicidal (Ae. aegypti, An. stephensi*, and *Cx. quinquefasciatus)*	Laboratory strains	20.10 18.40 21.84	[319]
59.	*Euphorbia hirta* Leaves (aqueous extract 10%)	$AgNO_3$ 1mM	NM	Spherical, 30–60 nm (SEM)	Larvicidal and pupicidal *(An. stephensi)*	Laboratory strains	10.14–27.89	[320]
60.	*Annona squamosa* Leaves (aqueous extract 5%)	$AgNO_3$ 1mM	Ratio 1:19 Incubation time: 4h Temperature: 95°C	irregular, 450 nm (SEM)	Larvicidal (*Ae. aegypti, An. stephensi,* and *Cx. quinquefasciatus*)	Laboratory strains	0.30 0.41 2.12	[321]
61.	*Rhizophora mucronata* Leaves (aqueous extract 10%)	$AgNO_3$ 1mM	Ratio 1:19 Incubation time: 10 min Temperature NM	Spherical, 60–95 nm (AFM)	Larvicidal (*Ae. aegypti* and *Cx. quinquefasciatus*)	Wild populations	0.59 0.89	[322]
62.	*Mimosa pudica* Leaves (aqueous extract 10%)	$AgNO_3$ 1mM	Ratio 88:12 Incubation time: 10 min Temperature NM	Spherical, 25– 60 nm (TEM)	Larvicidal *(An. subpictus and Cx. quinquefasciatus)*	Wild populations	13.90 11.73 52.01	[323]
63.	*Eclipta prostrate* Leaves (aqueous extract 10%)	$AgNO_3$ 1mM	Ratio 88:12 Incubation time NM Temperature NM	35–60 nm (SEM)	Larvicidal *(Cx. quinquefasciatus* and *An. subpictus)*	Wild populations	4.56 5.14	[324]
64.	*Nelumbo nucifera* Leaves (aqueous extract 10%)	$AgNO_3$ 1mM	Ratio 88:12 Incubation time: 10 min Temperature NM	spherical, triangle, and decahedral, 25– 80 nm (TEM)	Larvicidal *(An. subpictus* and *Cx. quinquefasciatus)*	Laboratory strains	0.69 1.10	[325]
65.	*Sida acuta* Leaves (aqueous extract 10%)	$AgNO_3$ 1mM	Ratio 12:88 Incubation time: 10 min	Spherical, triangle and decahedral, 18–35 nm (TEM)	Larvicidal *(An. stephensi Ae. aegypti,* and *Cx. quinquefasciatus)*	Wild populations	21.92 23.96 26.13	[326]
66.	*Wrightia tinctoria* leaves (aqueous extract 5%)	$CuSO_4.5H_2O$ NH_3	Ratio NM Incubation Time: 3 h Temperature NM	Spherical, 15–40 nm (TEM)	Larvicidal (*Ae. aegypti*)	Laboratory strains	32.10	[327]
67.	*Pouteria campechiana*, leaves (aqueous extract 15%)	$TiCl_4$ 1mM	Ratio 1:4 Incubation time: 24 h Temperature: 37°C	Spherical 73–140 nm (SEM)	Larvicidal and pupicidal (*Ae. aegypti*)	Laboratory strains	900	[328]
68.	*Coleus aromaticus* leaves (aqueous extract 10%)	$TiCl_4$ 1mM	Ratio 1:4 Incubation time: 24 h Temperature: 37°C	Spherical 16.5 nm (TEM)	Larvicidal (*Ae. aegypti*)	Laboratory strains	1000	[329]
69.	*Murraya koengii* leaves (aqueous extract 2.5%)	Na_2SeO_3 0,01M	Ratio 1:10 Incubation time: 24h Temperature: 37°C	Spherical 50–150 nm (TEM)	Larvicidal (*Ae. aegypti*)	Laboratory strains	3.54	[330]
70.	*Dillenia indica* leaves (aqueous extract 10%)	H_2SeO_3 10mM	Ratio 1:10 Incubation time: 24h Temperature: 37°C	Oval 248 nm (SEM)	Larvicidal (*Ae. aegypti,* and *Cx. quinquefasciatus*)	Laboratory strains	0.39 1.11	[331]
71.	*Cupressus sempervirens* leaves (aqueous extract 10%)	Na_2SeO_3 1mM	Ratio 3:22 Incubation time: 24h Temperature: 37°C	Spherical 11–55 nm (TEM)	Larvicidal (*Cx. pipiens*)	NM	28.25	[332]
72.	*Cuscuta reflexa* stem (aqueous extract 20%)	$Zn(NO_3)_2$ 0.1 M	Ratio NM Incubation time: 10 min Temperature: 60°C pH 9 (NaOH 0,2 M)	Spherical 40 nm (TEM, SEM)	Larvicidal *(An. stephensis)*	NM	50–250	[333]

73.	*Lawsonia inermis* leaf (aqueous extract 10%)	$Zn(NO_3)_2$ 1mM	Ratio NM Incubation time: 4 h Temperature 60°C	Irregular, 29– 43 nm (TEM, SEM)	Larvicidal *(An. stephensis*)	Wild populations	5,5–12,7	[334]
74.	*Lagenaria sicera* peel (aqueous extract 10 %)	$Zn(NO_3)_2$ 5 mM	Ratio 25:75	NM	Larvicidal *(An. stephensi*)	NM	56.46	[335]
75.	*Scadoxus multiflora* leaf (aqueous extract 30 %)	$Zn(CH_3COO)_2$. $2H_2O$ NM	Ratio 20:80 Incubation time: 3 h Temperature 60°C	Spherical, irregular 31 ± 2 nm (TEM, DLS)[j]	Larvicidal and ovicidal *(Ae. aegypti)*	Laboratory strains	120 120	[336]
76.	*Cipadessa baccifera* leaf (acetone, methanol, ethyl acetate, chloroform, petroleum benzene extracts 75 %)	$Zn(NO_3)_2$ 1 mM	Ratio NM Incubation time: 5 h Temperature: 65°C	Spherical 41.48 nm (TEM, SEM)	Larvicidal (*Cx. quinquefasciatus, An. stephensi*, and *Ae. aegypti)*	Laboratory strains	0.65–8.55	[337]
77.	*Annona squamosa,* fruit (aqueous extract 10%)	$Pd(OAc)_2$1mM	Ratio 1:8 Incubation time: 2h Temperature: 60°C	Spherical 80±5 nm (TEM)	Larvicidal (NM)	Laboratory strains	NM	[338]
78.	*Ocimum basilicum* leaves (aqueous extract 10%)	CdS NM	Ratio 1:1 Incubation time: 1h Temperature: 100°C	Spherical 3.84 nm (HR-TEM)	Larvicidal (*Cx. pipiens*)	Wild populations	11,3	[339]
79.	*Tagetes sp.,* petals (aqueous extract 1%)	$CdCl_2$ NM	Ratio 12:88 Incubation time:1h Temperature: NM	Spherical NM	Larvicidal (*Ae. albopictus*)	Laboratory strains	10	[340]
80.	*Uvaria chamae* leaves (aqueous extract 10%)	$FeCl_3$ 0.01M	Ratio 1:1 Incubation time: 10 min Temperature: room	Spherical 29–51 nm (SEM)	Larvicidal (*Cx. quinquefasciatus)*	Laboratory strains	NM	[341]
81.	*Acorus calamus,* rhizome (aqueous extract 10%)	$Fe_2(SO4)_3$ 1mM	Ratio 1:1 Incubation time: NM Temperature: room	Spherical 20 nm (TEM)	Larvicidal (*Cx. quinquefasciatus)*	Laboratory strains	30	[342]
82.	*Anthocepholus cadamba,* leaves (aqueous extract 5%)	$HAuCl_4$ NM	NM	Spherical, triangular, hexagonal, and rod, 20–50nm (TEM)	Larvicidal *(Cx. quinquefasciatus)*	Laboratory strains	1.08–21.82	[343]
83.	*Borassus aethiopum,* roots (aqueous extract 15%)	$NiCl_2.6H_2O$ 10mM/$AgNO_3$ 10mM	Ratio 1:5 Incubation time: 30 min Temperature: 80°C	Size NM Shape NM	Larvicidal (*Cx. quinquefasciatus)*	Wild population	5.73	[344]
84.	*Borassus flabellifer,* roots (aqueous extract 4%)	$CoCl_2$/$AgNO_3$ 1mM	Ratio 1:5 Incubation time: 24h Temperature: 37°C	Size NM Shape NM	Larvicidal (*Cx. quinquefasciatus)*	Wild populations	5.24–13.63	[345]

[a]LC_{50}: 50 % lethal concentration (in ppm unless otherwise stated) [b]NM: not mentioned [c]SEM: Scanning Electron Microscopy [d]EDX: Energy Dispersive X-Ray Spectroscopy [e]TEM: Transmission Electron Microscopy [f]FE-SEM: Field Emission Scanning Electron Microscopy [g]EDS: Energy Dispersive Spectroscopy [h]HR-TEM: High-Resolution Transmission Electron Microscopy [i]AFM: Atomic Force Microscopy [j]DLS: Dynamic Light Scattering
